# Poverty, Disease, and Crime

**Published:** 1859-02

**Authors:** 


### POVERTY, DISEASE, AND CRIME,

Go together; so do thrift, health, and good citizenship. The panacea for human sorrow is not the removal of poverty. That will not reach the root of the evil. Make a child good, and you give good assurance against idleness, beggary, and wasting disease. Teach a child to be clean, to be truthful, to hate all wrong doing, to be industrious and saving, and with a thorough education in "reading, writing, and arithmetic," you make him rich beyond the inheritance of paternal millions. Poverty is neither a curse nor a crime. Had we the peopling of a world like this, with present views of human nature and human need, we would turn every son and daughter into the great harvest field of life without a shirt to the back or an implement to the hand. The necessity for "device" has been the material salvation of the human family. No children are so utterly worthless as those who never knew an obstacle between an expressed desire and its gratification. No child is so irretrievably ruined as the one whose parent is its slave. Let every one enter the world with an income, and it would, under the present constitution of things, become, within a century, a world of idleness, gluttonny, and havoc-making disease; so that while it is true that, in one sense of the word, "the destruction of the poor is their poverty," it is, in another sense, not less demonstrable, that poverty is the material safety of the race—as witness the brightest, highest names in history, ancient or modern. Poverty has been the main stimulus in almost all sublime lives; at the same time, it goads men to the commission of the gravest crimes. What makes the difference? Not certainly what we call " intelligence," mere " education," about which unbalanced minds so constantly prate, as an infallible cure for human woe, the certain means of human weal.

Mere " education," in the common acceptance of the term, makes a man a better saint or a bigger devil, according to the direction taken in the outset; and that direction is the result of the instillation, or its neglect, *from the first year of life*, of those principles of human conduct imparted by actions as well as words, and which are founded in " love, joy, peace, long suffering, gentleness, goodness, faith, meekness, temperance;" for " against such there is no law." Let the reader go over all the qualities just named, and consider for a moment how not one of them is inseparable from the character of a gentleman and an honest man; and, if all were such, it is easy to see that this would be a world of thrift, of enjoyment, and elevation. If, therefore, the words quoted are interpreted aright, they mean that in proportion as men follow out in their daily conduct the great principles of love, goodness, and temperance, however limited may be their " education," they escape human suffering for all time, as far as that may arise from causes within themselves. The surest way, therefore, to beatify the human race permanently, is not to begin at the half-way house, by endeavoring to banish poverty and existing disease. We must begin at the beginning, and make men good by diligently sowing the seeds of " love," and " goodness," and " temperance," while yet in early infancy. This high, holy, and important duty, belongs to parents, and ought to be delegated to no others. But the fashion of the times—and one most widely prevalent—is to turn over this first of all duties to Sunday school teachers, many of whom are in their teens, and not a few personally ignorant of the " great salvation."

As far as the children of professing Christians are concerned, and as far as Sunday schools, as now too generally conducted practically, take the early religious instruction of children in the distinctive sentiments of their faith out of the parents' hands, and commit it to the unfledged, who themselves need to be taught, it were better that they, as now generally conducted, and as to their tendencies in relation to the children of the church, had never been heard of.

"A thorough education," a "superior education" of all young people, is not the panacea for the world's ills; will never free it from destitution, crime, disease, and premature death, using these terms in their general accepted sense. We must go behind the school teacher, because the child's destiny is shaped before it enters the A B C school-room; direction is given to its goings-out, to a very great extent, before it leaves its mother's lap, and while yet it is toddling about the floor and amusing itself with its toys; and among the first things may be mentioned frankness, truthfulness, consistency, and affection. If an infant sees these in its parents, day by day, in all things, it will grow up to be like them with encouraging certainty, paving the way for a parental influence in teachings higher and still more important, which will form the character in such a mould as will make it safe for all time.

Father and mother are equally bound to do all within their power in forwarding these primary educations; but as the mother is always at home, and possesses the warmer and more entire affection and confidence of the child, a higher share of the responsibility rests on her; and as over her the clergyman who preaches to her every Sabbath has a commanding influence, we come back to the two first truths. First—

The clergy of all denominations must wake up to a greater diligence in urging mothers to an imitation of Hannah of old, whose concern began before little Samuel saw the light of day, and which concern never flagged, until he was officially committed to the temple. Mothers should be taught that the bedewing influence of meditative piety should be shed on the child's nature when "as yet it is not," and they should be urged unceasingly to follow it up day by day, until the character is fully formed. To do all this properly, mothers, amid the toils and trials and discouragements of daily life, need counsel, and sympathy, and help from the minister—given, not from the stately pulpit, but from the daily greeting and the friendly fireside call, where there is a felt confidence and a felt sympathy, the imparting and the reception of which are both happifying.

Thus acting, the clergyman of an ordinary congregation would, with other necessary duties, have his time fully employed. Second—to do that, others should see to it that his temporal wants are promptly, fully, and liberally met, and this devolves on the people of his charge. In short, the only hope of a world's permanent redemption from crime and disease is in a faithful ministry, well paid by the people, to enable them to give their whole time to the care of the flock over which they are the shepherds. And to make a beginning, let the reader lay down this page and rest not until he has done all he could to secure for his minister an abundant support; nor rest here. If that minister fails of an entire consecration of himself to the faithful performance of what has been marked out, turn him out as unworthy of his hire, and even if in all things else he be a very Gabriel.

We may as well wake up to the fact first as last, that all modes of "reform" of human elevation will fail, which are anything short of preventives, and that efforts for the amelioration of the condition of mankind, to be permanently successful, must reach behind the college, the academy, the Sunday school, they must reach to the infant child—must go before its birth—must operate through a mother's prayers and tears, and bedewing piety. The Editor hopes that abler minds will carry out the idea, the subject having been suggested by a letter from a rich man, without family, who desires to lay out some scores of thousands of dollars in a manner which shall most certainly accomplish the highest results. He has already spent much time and large sums of money in diffusing information which was calculated to benefit the masses, and especially the poor. Having been the architect of his own fortunes, he has not, in his social and pecuniary elevation, forgotten those who are now enduring that grinding poverty through which he once passed himself, and knowing its hardships, its temptations, and its trials, he has a heart broad and full enough to do something to save others from them, and we do certainly believe that his objects will be most radically and permanently secured by a faithful ministry and a faithful motherhood:

"Dr. Hall:

"Dear Sir—We are advised to 'take *time by the forelock!*' You are evidently engaged in the endeavor to instruct the masses to take *disease by the forelock.* Why, then, may we not endeavor to teach the masses how to take '*poverty*' by the forelock? But first we must determine its cause or causes.

"William Penn said—'If you would reform the world, you must begin the reformation with your children.' (Not mine, for I ar'nt got any!) I contend that one great cause—if not the principal cause of poverty—arises from the fact that children are taught from their infancy to be spendthrifts, fearful that the little dears will not know, when arrived at the years of maturity, *how to spend money economically!* and, therefore, they are taught to spend all they get, and as fast as they get it. I should say that children should be taught how to save money, and that to spend it is as much a sin as to *lie* or *steal*, and, if there is any spending to be done, let it be done by the parent. This is my doctrine, and I would pay a handsome trifle for a good essay upon this subject.

"My worthy pa used to say—'The destruction of the poor is their poverty!' Many a one has been destroyed by consumption; but this is only the *effect*, and so is poverty only an effect. Let us have the cause, that the effect may be averted. If you agree with me, I should be pleased to see an article in your journal upon this subject; but if not, we will drop the subject like a hot potato, and let it slide.

"By the way, doctor, I have had one of your 'physiological chairs' made (ten-inch seat, not eight, as you suggest), and it gives so much and so general satisfaction, that I have ordered several more made.

"Mr. Fowler took a seat, and pronounced it a capital idea.

"Yours, "C."

Let the three points of our article remain distinctly before the reader's mind. First—That mere education, talent, genius, is not sufficient to restrain men from crime, else Lord Bacon would never have been bribed, Dr. Dodd would never have perpetrated a forgery—else Voltaire might have been a Luther, Hume a Calvin, and Apollyon a Gabriel. Dr. Murray says, with great truth: "High talent, unless early cultivated, as was that of Moses, and Milton, and Baxter, and Edwards, and Wesley, and Robert Hall, is the most restive under moral restraints; is the most fearless in exposing itself to temptation; is the most ready to lay itself on the lap of Delilah, trusting in the lock of its strength. And, alas! like Sampson, how often is it found blind and grinding in the prison house, when it might be wielding the highest political power, or civilising and evangelising the nations."

Second—The best time for making the imprint for eternity on an immortal nature is while it is yet in its mother's womb. It was while bearing the unborn Napoleon, that the mother scoured the country at the side of her warrior husband. It was before the birth of Samuel, who became higher than kings, that Hannah sanctified him in her heart, set him apart, and consecrated him to a religious life.

Third—It was Eli the priest who comforted Hannah in her despondency, and the priests were so amply cared for, that they could give their whole time to their duties.

